# Supplementary material for: Evaluating the risk of conflict on recent Ebola outbreaks in Guinea and the Democratic Republic of the Congo
Source: BMC Public Health. 2024 Mar 20;24:860. doi: 10.1186/s12889-024-18300-8 (PMC10953285; doi:10.1186/s12889-024-18300-8)
Supplement: Supplementary file 4 — Supplementary Material 4. [file 12889_2024_18300_MOESM4_ESM.pdf]

Additional file 4

Proximity of battles, protests and riots to major cities in Guinea and the DRC

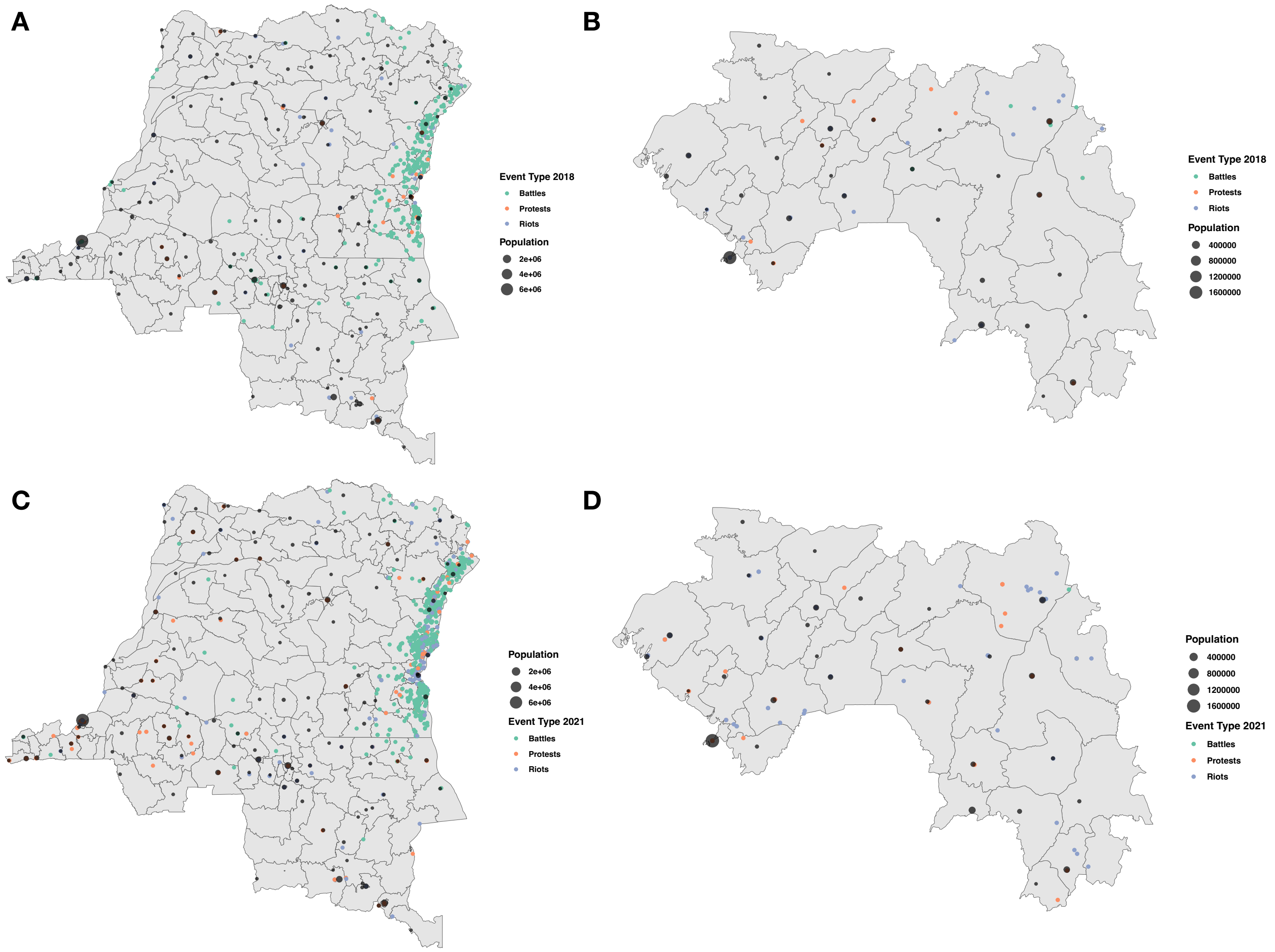

Fig.S2 The location, by longitude and latitude, of major cities (point size = population size) and conflict event for **A**, the Democratic Republic of Congo in 2018, **B**, Guinea in 2018, **C**, the Democratic Republic of Congo in 2021 and **D**, Guinea in 2021.
